# Prognostic Value of the Cumulative Inflammatory Index (IIC) in Patients with Non-ST-Segment Elevation Myocardial Infarction

**DOI:** 10.3390/biomedicines14071415

**Published:** 2026-06-23

**Authors:** Yakup Yiğit, Abdulmecit Afşin, Güney Sarioğlu, Kadir Uçkaç

**Affiliations:** Department of Cardiology, Faculty of Medicine, Malatya Turgut Özal University, 44090 Malatya, Türkiye

**Keywords:** myocardial infarction, Cumulative Inflammatory Index, inflammation, mortality, NSTEMI

## Abstract

**Background/Objectives:** Inflammation plays a central role in the pathophysiology and prognosis of non-ST-segment elevation myocardial infarction (NSTEMI). This study aimed to investigate the clinical and prognostic significance of the Cumulative Inflammatory Index (IIC) in patients with NSTEMI. **Methods:** This single-center, retrospective study included 2274 individuals, comprising 1172 patients with NSTEMI and 1102 angiographic controls without acute coronary syndrome or obstructive coronary artery disease. IIC was calculated using mean corpuscular volume, red cell distribution width, neutrophil count, and lymphocyte count. The primary outcome was 360-day all-cause mortality in the NSTEMI cohort. Logistic regression, receiver operating characteristic curve analysis, and DeLong testing were performed. **Results:** Patients with NSTEMI had significantly higher IIC values than controls [9.08 (4.05–15.03) vs. 1.90 (1.45–2.89), *p* < 0.001]. Among NSTEMI patients, non-survivors had significantly higher IIC levels than survivors [14.25 (8.56–26.59) vs. 8.57 (3.73–14.06), *p* < 0.001]. In multivariable logistic regression analysis, IIC remained independently associated with 360-day all-cause mortality after adjustment for age, diabetes mellitus, estimated glomerular filtration rate, hemoglobin, albumin, and C-reactive protein (OR: 1.045, 95% CI: 1.029–1.060; *p* < 0.001). IIC showed a modestly higher area under the curve among the evaluated indices (AUC: 0.704). **Conclusions:** IIC was significantly elevated in patients with NSTEMI and was independently associated with 360-day all-cause mortality. IIC may serve as a simple adjunctive marker for risk stratification in patients with NSTEMI.

## 1. Introduction

Acute coronary syndrome (ACS) is one of the leading causes of cardiovascular morbidity and mortality worldwide. While current diagnostic and treatment strategies have significantly improved the management of NSTEMI, early diagnosis of patients at high risk of mortality remains clinically important. Current risk assessment is primarily based on clinical presentation, electrocardiographic findings, cardiac biomarkers, comorbidities, renal function, and established risk scores; however, additional simple and widely available biomarkers could improve prognostic assessment in daily practice [1,2].

Inflammation plays a significant role in the formation, progression, and instability of atherosclerotic plaques. Inflammatory activation leads to endothelial dysfunction, plaque rupture or erosion, thrombus formation, microvascular disruption, and subsequent myocardial damage. The inflammatory hypothesis of atherothrombosis is supported by experimental and observational data, as well as clinical trial evidence showing that targeted anti-inflammatory therapy can reduce recurrent cardiovascular events in selected patients with a history of myocardial infarction and a high inflammatory burden [3,4,5]. In this sense, inflammatory biomarkers are gaining increasing importance in the evaluation of patients with ACS.

Among these markers, indices derived from complete blood counts are particularly attractive because they are inexpensive, readily available, and routinely measured upon hospital admission. The neutrophil-lymphocyte ratio (NLR) reflects the balance between innate inflammatory activation and adaptive immune response, while the platelet-lymphocyte ratio (PLR) integrates thrombotic and inflammatory pathways. Recently, composite indices such as the systemic immune-inflammation index (SII) and pan-immune-inflammation value (PIV) have been proposed to provide a broader assessment of systemic inflammation by integrating neutrophil, lymphocyte, platelet, and monocyte counts. Previous studies and meta-analyses indicate that high NLR, PLR, SII, and PIVs are associated with adverse cardiovascular outcomes in patients with ACS and myocardial infarction [6,7,8,9,10].

In addition to the inflammatory indices mentioned above, erythrocyte-related parameters can also provide prognostic information in cardiovascular disease. Red blood cell distribution width (RDW), a measure of erythrocyte size variability, has been associated with inflammation, oxidative stress, impaired erythropoiesis, nutritional status, and adverse clinical outcomes. In patients with ACS without ST segment elevation, high RDW is thought to be associated with increased long-term mortality. Mean corpuscular volume (MCV), another routinely measured erythrocyte parameter, can also be affected by systemic inflammation, nutritional status, and bone marrow response. These observations suggest that integrating erythrocyte indices with leukocyte-based inflammatory parameters may provide a more comprehensive measure of systemic inflammatory burden [11,12].

The IIC is a relatively new composite hematological inflammatory index calculated using MCV, RDW, neutrophil count, and lymphocyte count. It was previously introduced together with the mean corpuscular volume-to-lymphocyte ratio in patients with acute pancreatitis and has subsequently been evaluated in other inflammatory conditions [13,14]. The rationale for IIC is based on the integration of leukocyte-derived inflammatory activation with erythrocyte-related parameters. Neutrophils and lymphocytes reflect innate inflammatory activation and adaptive immune status, respectively, whereas RDW reflects erythrocyte heterogeneity associated with inflammation, oxidative stress, and impaired erythropoiesis. MCV may provide additional information on erythrocyte size and bone marrow response, which can be influenced by systemic inflammation, nutritional status, and chronic disease. Therefore, combining MCV with RDW and leukocyte parameters may provide a broader hematological reflection of systemic inflammatory burden than RDW or NLR alone [13,14]. However, whether MCV adds prognostic information beyond RDW alone in patients with NSTEMI remains unclear. Given the close relationship between NSTEMI, systemic inflammation, plaque instability, and adverse long-term outcomes, IIC may have potential value as a simple and accessible adjunctive risk marker. Accordingly, the present study aimed to evaluate the prognostic significance of IIC in NSTEMI and to compare its discriminatory performance with established hematological inflammatory indices.

## 2. Materials and Methods

This single-center, retrospective, observational study included patients evaluated at Malatya Training and Research Hospital. Patients evaluated between January 2022 and January 2025 were retrospectively screened. The study was designed to assess the clinical and prognostic significance of the IIC in patients with non-ST-segment elevation myocardial infarction (NSTEMI). Initially, a total of 2700 patients were screened. After applying pre-defined exclusion criteria and excluding patients who lacked the necessary laboratory data for calculating inflammatory indices or for whom 360-day mortality data were unavailable, a total of 2274 individuals were included in the final analysis. Complete-case analysis was performed, and no imputation was used for missing data. Of these, 1172 patients constituted the NSTEMI group, and 1102 patients constituted the control group. The control group consisted of patients who underwent coronary angiography during the same study period for suspected coronary artery disease or chest pain, but who had no evidence of acute coronary syndrome and no obstructive coronary artery disease. Acute coronary syndrome was excluded in the control group based on clinical evaluation, electrocardiographic findings, and cardiac troponin measurements. Angiographic controls were selected instead of healthy individuals to obtain a clinically more comparable reference group with similar cardiovascular risk factors and comorbidities, while excluding acute coronary syndrome and obstructive coronary artery disease by coronary angiography. Non-obstructive coronary artery disease was defined as the absence of ≥50% luminal stenosis in any major epicardial coronary artery on coronary angiography.

NSTEMI was diagnosed according to current clinical criteria, including ischemic symptoms, electrocardiographic findings without persistent ST segment elevation, and a rise and/or fall in cardiac troponin with at least one value above the 99th percentile upper reference limit, together with evidence of myocardial ischemia.

The study consisted of two main analytical phases. First, demographic characteristics, clinical comorbidities, laboratory parameters, and hematological inflammatory indices were compared between the NSTEMI and control groups. All laboratory parameters were obtained from admission blood samples collected before the initiation of medical therapy. Second, the NSTEMI cohort was analyzed separately to determine the relationship between inflammatory indices and 360-day all-cause mortality.

### 2.1. Inclusion and Exclusion Criteria

Patients aged ≥18 years with a confirmed diagnosis of NSTEMI and available baseline complete blood count parameters were included in the NSTEMI group. The control group included patients aged ≥18 years who underwent coronary angiography for suspected coronary artery disease or chest pain during the same period, but who had no clinical, electrocardiographic, biochemical, or angiographic evidence of acute coronary syndrome or obstructive coronary artery disease.

Exclusion criteria were ST-segment elevation myocardial infarction, active infection, active malignancy, known hematological disease, chronic alcohol use, chronic liver disease or severe hepatic dysfunction, chronic inflammatory or autoimmune disease, recent major surgery or trauma, corticosteroid or immunosuppressive therapy, and missing laboratory data required for the calculation of hematological inflammatory indices. In the control group, patients with acute coronary syndrome, obstructive coronary artery disease, or missing angiographic data were excluded. NSTEMI patients without available 360-day all-cause mortality data were excluded from the prognostic analysis.

### 2.2. Data Collection

All data were retrospectively obtained from the hospital electronic medical record system. Demographic characteristics, comorbid conditions, laboratory findings, survival status, and coronary angiography reports were recorded. Coronary angiography reports were reviewed to verify the absence of obstructive coronary artery disease in the control group. The following clinical variables were collected: age, sex, hypertension, diabetes mellitus, chronic kidney disease, and 360-day all-cause mortality. Laboratory parameters included leukocyte count, neutrophil count, lymphocyte count, monocyte count, hemoglobin, platelet count, red cell distribution width, mean corpuscular volume, aspartate aminotransferase, alanine aminotransferase, albumin, glucose, estimated glomerular filtration rate, potassium, sodium, lactate dehydrogenase, C-reactive protein, CK-MB mass, and troponin I levels. Biochemical parameters were analyzed using the Siemens Atellica Solution system (Siemens Healthcare Diagnostics Inc., Tarrytown, NY, USA). No commercial cell lines, experimental samples, or additional reagents/materials were used in this retrospective observational study.

### 2.3. Calculation of Hematological Inflammatory Indices

All hematological inflammatory indices were calculated from admission laboratory parameters. The formulas were as follows:

$$\begin{array}{c} NLR=neutrophil\;count/lymphocyte\;count;\\ PLR=platelet\;count/lymphocyte\;count;\\ SII=platelet\;count\times neutrophil\;count/lymphocyte\;count;\\ PIV=neutrophil\;count\times platelet\;count\times \mathrm{monocyte}\;\mathrm{count}/\mathrm{lymphocyte}\;\mathrm{count};\\ IIC=(MCV\times RDW\times neutrophil\;count)/(lymphocyte\;count\times 1000). \end{array}$$

Mean corpuscular volume, red cell distribution width, and absolute neutrophil, lymphocyte, monocyte, and platelet counts were obtained from complete blood count parameters.

### 2.4. Study Outcomes

The primary outcome of the study was 360-day all-cause mortality in patients with NSTEMI. Secondary outcomes included the comparison of inflammatory indices between the NSTEMI and control groups, the evaluation of clinical and laboratory differences between surviving and non-surviving NSTEMI patients, and the assessment of the prognostic value of IIC for 360-day all-cause mortality.

### 2.5. Statistical Analysis

Statistical analyses were performed using SPSS software version 26.0 (IBM Corp., Armonk, NY, USA). The normality of continuous variables was assessed using the Kolmogorov–Smirnov or Shapiro–Wilk test, together with visual inspection of histograms and Q-Q plots. Normally distributed continuous variables were expressed as mean ± standard deviation, whereas non-normally distributed variables were expressed as median and interquartile range. Categorical variables were presented as numbers and percentages.

Continuous variables were compared using the independent-samples *t*-test or Mann–Whitney U test, according to data distribution. Categorical variables were compared using the Pearson chi-square test or Fisher’s exact test, as appropriate. The same statistical approach was applied for comparisons between surviving and non-surviving patients within the NSTEMI cohort.

Univariable logistic regression analysis was performed to identify variables associated with mortality. Because exact time-to-event data were not available, logistic regression analysis was used to evaluate the association between inflammatory indices and mortality. Multivariable logistic regression models were then constructed to evaluate whether hematological inflammatory indices were independently associated with mortality after adjustment for clinically relevant covariates, including age, diabetes mellitus, estimated glomerular filtration rate, hemoglobin, albumin, and C-reactive protein. Since NLR, PLR, SII, PIV, and IIC are mathematically and biologically interrelated, each inflammatory index was entered into a separate multivariable model to reduce multicollinearity. Odds ratios and 95% confidence intervals were reported. For PLR, SII, and PIV, odds ratios were expressed per 100-unit increase.

Receiver operating characteristic curve analysis was performed to assess the discriminatory performance of inflammatory indices for predicting mortality. The area under the curve, 95% confidence interval, optimal cut-off value, sensitivity, specificity, and Youden index were calculated. AUC values were compared using the DeLong test. A two-sided *p*-value of <0.05 was considered statistically significant.

### 2.6. Ethical Considerations

This retrospective study was approved by the Health Sciences Scientific Research Ethics Committee of Malatya Turgut Özal University, Malatya, Türkiye, with application number 2026/176. The study was approved unanimously as ethically appropriate at the meeting dated 7 May 2026, session number 23. The official approval document was issued on 13 May 2026, with document number E-30785963-020-391939. The study was conducted in accordance with the principles of the Declaration of Helsinki. Because of the retrospective design of the study and the use of anonymized data obtained from hospital records, individual informed consent was not required.

## 3. Results

### 3.1. Baseline Clinical Characteristics

A total of 2274 individuals were included in the study, comprising 1172 patients with NSTEMI and 1102 controls without acute coronary syndrome or obstructive coronary artery disease. Age, sex distribution, and the prevalence of diabetes mellitus, hypertension, and chronic kidney disease were comparable between the Control and NSTEMI groups. (Table 1).

### 3.2. Laboratory Parameters and Hematological Inflammatory Indices

Laboratory parameters differed significantly between the control and NSTEMI groups. Patients with NSTEMI had higher leukocyte, neutrophil, and monocyte counts, whereas lymphocyte count was significantly lower than in the control group. Hemoglobin and albumin levels were significantly lower in the NSTEMI group, while RDW, MCV, AST, ALT, glucose, LDH, and CRP levels were significantly higher. In addition, eGFR, potassium, and sodium levels were significantly lower in patients with NSTEMI. 

All hematological inflammatory indices were significantly elevated in the NSTEMI group. Median NLR was higher in NSTEMI patients than in controls [7.23 (3.25–11.86) vs. 1.67 (1.27–2.37), *p* < 0.001]. Similarly, PLR [182.15 (110.13–286.29) vs. 107.57 (84.81–141.96), *p* < 0.001], SII [1701.12 (752.29–2874.68) vs. 432.22 (312.87–633.77), *p* < 0.001], PIV [924.02 (385.15–1930.10) vs. 234.67 (147.97–374.95), *p* < 0.001], and IIC [9.08 (4.05–15.03) vs. 1.90 (1.45–2.89), *p* < 0.001] were markedly increased in the NSTEMI group (Table 2).

### 3.3. Mortality in NSTEMI Patients

Among patients with NSTEMI, 1009 patients survived and 163 (13.9%) patients died within 360 days. Non-survivors were significantly older than survivors (77.10 ± 12.38 vs. 70.04 ± 12.86 years, *p* < 0.001). The prevalence of hypertension and chronic kidney disease was significantly higher among non-survivors. Sex distribution and diabetes mellitus prevalence did not differ significantly between survivors and non-survivors.

Non-survivors had higher leukocyte and neutrophil counts, lower lymphocyte count, lower hemoglobin, lower eGFR, lower sodium, lower albumin, and higher potassium, LDH, ALT, and CRP levels compared with survivors. RDW was also significantly higher in non-survivors [14.60 (13.80–16.70) vs. 13.80 (13.10–15.20), *p* < 0.001]. No significant differences were observed in CK-MB mass, troponin I, monocyte count, MCV, platelet count, AST, or glucose levels.

All hematological inflammatory indices were significantly higher in non-survivors than in survivors. Median NLR was 10.34 (6.40–20.13) in non-survivors and 6.73 (3.03–11.34) in survivors (*p* < 0.001). Similarly, PLR, SII, PIV, and IIC were significantly elevated in non-survivors. Median IIC was 14.25 (8.56–26.59) in non-survivors compared with 8.57 (3.73–14.06) in survivors (*p* < 0.001) (Table 3).

### 3.4. Univariable Logistic Regression Analysis

In univariable logistic regression analysis, age, eGFR, hemoglobin, albumin, CRP, NLR, PLR, SII, PIV, and IIC were significantly associated with 360-day all-cause mortality in patients with NSTEMI. Older age was associated with increased mortality risk (OR: 1.045, 95% CI: 1.030–1.060; *p* < 0.001), whereas higher eGFR, hemoglobin, and albumin levels were associated with lower mortality risk.

Among hematological inflammatory indices, NLR (OR: 1.074, 95% CI: 1.054–1.094; *p* < 0.001), IIC (OR: 1.058, 95% CI: 1.043–1.073; *p* < 0.001), PIV, SII, and PLR were significant predictors of 360-day all-cause mortality. Male sex, diabetes mellitus, and troponin I were not significantly associated with 360-day all-cause mortality in univariable analysis (Table 4).

### 3.5. Multivariable Logistic Regression Analysis

Multivariable logistic regression analyses were performed using separate models for each hematological inflammatory index. After adjustment for age, diabetes mellitus, eGFR, hemoglobin, albumin, and CRP, all inflammatory indices remained independently associated with 360-day all-cause mortality.

In Model 1, NLR was an independent predictor of 360-day all-cause mortality (OR: 1.058, 95% CI: 1.037–1.080; *p* < 0.001). In Model 2, PIV remained independently associated with mortality (OR: 1.010, 95% CI: 1.002–1.018; *p* = 0.017). In Model 3, SII was independently associated with mortality (OR: 1.015, 95% CI: 1.008–1.022; *p* < 0.001). In Model 4, IIC was an independent predictor of 360-day all-cause mortality (OR: 1.045, 95% CI: 1.029–1.060; *p* < 0.001). In Model 5, PLR was also independently associated with mortality (OR: 1.163, 95% CI: 1.066–1.269; *p* = 0.001) (Table 5). Goodness-of-fit analyses showed acceptable model fit for all multivariable logistic regression models. The Hosmer–Lemeshow test was non-significant for all models, and detailed goodness-of-fit statistics are presented in Supplementary Table S1.

### 3.6. ROC Curve Analysis and Comparison of Predictive Performance

**Figure 1:**
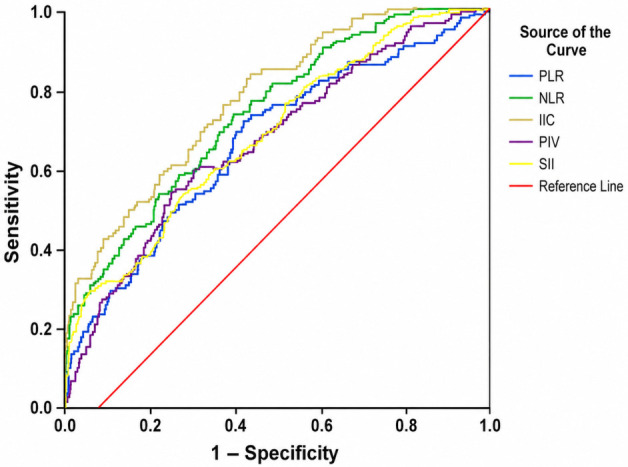
ROC curve analysis of inflammatory indices for predicting 360-day all-cause mortality. ROC, receiver operating characteristic; PLR, platelet-to-lymphocyte ratio; NLR, neutrophil-to-lymphocyte ratio; IIC, Cumulative Inflammatory Index; PIV, pan-immune-inflammation value; SII, systemic immune-inflammation index.

ROC curve analysis was performed to evaluate the discriminatory ability of hematological inflammatory indices for predicting 360-day all-cause mortality. Among all indices, IIC showed a modestly higher AUC value (AUC: 0.704, 95% CI: 0.661–0.746; *p* < 0.001). The AUC values were 0.676 for NLR, 0.636 for SII, 0.621 for PIV, and 0.619 for PLR, all with *p* < 0.001. The optimal cut-off value of IIC for predicting 360-day all-cause mortality was ≥8.20, with 81.5% sensitivity, 67.9% specificity, and a Youden index of 0.494. The optimal cut-off values were ≥6.93 for NLR, ≥170 for PLR, ≥1304 for PIV, and ≥2203 for SII (Table 6). The corresponding ROC curves are shown in Figure 1. In bootstrap internal validation with 1000 resamples, the discriminatory performance of IIC remained stable. The bootstrap-validated AUC for IIC was 0.706, with a 95% confidence interval of 0.663–0.748.

Pairwise DeLong comparisons demonstrated that IIC had significantly better discriminatory performance than NLR, SII, PIV, and PLR. The AUC difference was 0.028 for IIC versus NLR, 0.067 for IIC versus SII, 0.083 for IIC versus PIV, and 0.084 for IIC versus PLR, with all comparisons reaching statistical significance (Table 7).

## 4. Discussion

In the present study, we evaluated the clinical and prognostic significance of the IIC in patients with NSTEMI. The main findings were as follows: first, NSTEMI patients had significantly higher IIC values and other hematological inflammatory indices compared with controls without acute coronary syndrome or obstructive coronary artery disease; second, among NSTEMI patients, non-survivors had markedly higher IIC levels than survivors; third, IIC remained independently associated with 360-day all-cause mortality after adjustment for age, diabetes mellitus, eGFR, hemoglobin, albumin, and CRP; and fourth, IIC showed a modestly higher discriminatory performance among the evaluated inflammatory indices, outperforming NLR, PLR, SII, and PIV in pairwise DeLong comparisons. The retrospective single-center design limits the external validity and generalizability of our findings. Therefore, the results should be interpreted cautiously and validated in prospective multicenter cohorts.

Risk stratification in NSTEMI remains an essential component of clinical management. Established risk models, such as the GRACE score, provide clinically useful prognostic information using readily available variables including age, hemodynamic status, renal function, cardiac biomarkers, and electrocardiographic findings [15]. However, these scores do not directly incorporate cellular inflammatory activity. This is clinically relevant because inflammation is a central mechanism in atherosclerotic plaque progression, plaque destabilization, thrombus formation, and adverse post-infarction remodeling [16]. In addition, randomized clinical trials showing cardiovascular benefit with anti-inflammatory strategies have further strengthened the concept that inflammation is not merely an epiphenomenon but an active contributor to residual cardiovascular risk [17,18].

Complete blood count-derived inflammatory indices have attracted attention because they are inexpensive, rapidly available, and easily reproducible. In the present study, NLR, PLR, SII, PIV, and IIC were all significantly higher in NSTEMI patients than in controls and were also associated with 360-day all-cause mortality. These findings are consistent with previous studies showing that higher NLR predicts adverse outcomes in patients with acute coronary syndrome [19], while PLR has been associated with long-term mortality after non-ST-elevation myocardial infarction [20]. Similarly, SII has been reported to predict adverse clinical outcomes in patients with coronary artery disease, and PIV has recently been linked to coronary artery disease severity in NSTEMI patients [21,22].

The distinctive feature of IIC is that it integrates both leukocyte-derived and erythrocyte-derived inflammatory information. While NLR mainly reflects the balance between neutrophil-mediated innate inflammation and lymphocyte-related immune regulation, IIC additionally incorporates MCV and RDW. RDW has been associated with adverse outcomes after acute myocardial infarction and may reflect inflammation, oxidative stress, impaired erythropoiesis, and nutritional or metabolic abnormalities [23]. Therefore, the higher prognostic performance of IIC in our study may be explained by its broader biological structure, combining neutrophil activation, lymphopenia, and erythrocyte heterogeneity into a single index. However, because IIC includes erythrocyte-related parameters, its biological interpretation should be cautious. RDW and MCV may be influenced not only by inflammation but also by anemia, renal dysfunction, nutritional deficiencies, and chronic systemic conditions. Although adjustment was performed for hemoglobin, albumin, eGFR, and CRP, residual confounding from unmeasured nutritional and hematologic factors may remain.

In ROC analysis, IIC demonstrated a modestly higher AUC value among all evaluated indices. The optimal IIC cut-off value showed high sensitivity and moderate specificity for predicting 360-day all-cause mortality. Therefore, IIC should not be interpreted as a stand-alone prognostic tool. Rather, it may serve as a practical screening marker to identify NSTEMI patients who may require closer clinical attention. Its low cost and availability from routine admission blood tests may support its use as an adjunctive marker for early risk assessment.

In DeLong comparisons, IIC showed statistically higher discriminatory performance than NLR, PLR, SII, and PIV. However, the absolute AUC difference between IIC and NLR was small. Therefore, the clinical relevance of this improvement may be limited and should be interpreted cautiously. This suggests that incorporating erythrocyte indices into leukocyte-based inflammatory assessment may improve risk discrimination. Recent non-cardiovascular studies have also investigated IIC and related MCV-based inflammatory markers in inflammatory diseases, supporting the concept that erythrocyte parameters may provide clinically relevant information beyond conventional leukocyte ratios [24]. Nevertheless, the use of IIC in NSTEMI remains relatively unexplored, and our findings should be interpreted as hypothesis-generating until externally validated.

This study has several limitations. First, the study was designed as a retrospective and single-center study; therefore, selection bias and unmeasured confounding cannot be completely excluded. Although the control group consisted of angiographic comparators rather than healthy individuals, residual differences in clinical characteristics between groups may still have influenced inflammatory indices. Second, the primary endpoint was 360-day all-cause mortality. Cardiovascular mortality could not be assessed. While all-cause mortality is clinically significant, it is a non-specific endpoint and may also reflect non-cardiovascular causes of death, particularly in elderly patients with chronic comorbidities. Therefore, the relationship between IIC and mortality should be interpreted as a correlation with the 360-day all-cause mortality rate, and not as a specific predictor of cardiovascular death or adverse events associated with NSTEMI. Because exact time-to-event data and censoring times were not consistently available, Cox proportional hazards regression and Kaplan–Meier survival analysis could not be performed. Therefore, logistic regression was used to evaluate 360-day all-cause mortality as a binary endpoint. This approach may limit prognostic interpretation because it does not account for the timing of death and does not allow censoring to be modeled as in conventional survival analyses. Accordingly, the association between IIC and mortality should be interpreted as a relationship with 360-day mortality status rather than as a time-dependent survival risk. Cardiovascular mortality, recurrent myocardial infarction, heart failure hospitalization, stroke, and target vessel revascularization could not be evaluated separately. Third, the inflammatory indices were calculated only from baseline laboratory parameters obtained at hospital admission. Serial changes in IIC, NLR, PLR, SII, and PIV during hospitalization or follow-up were not assessed. Fourth, left ventricular ejection fraction, GRACE/TIMI risk scores, detailed coronary lesion severity, treatment strategy, and discharge medications were not included in the models because these variables were not consistently available in the retrospective dataset. In addition, Killip class, hemodynamic status and culprit vessel were not consistently available. Because these NSTEMI-related variables may influence 360-day mortality, residual confounding cannot be excluded.

## 5. Conclusions

In conclusion, the present study demonstrated that IIC was significantly elevated in patients with NSTEMI compared with controls and was independently associated with 360-day all-cause mortality. Compared with other hematological inflammatory indices, including NLR, PLR, SII, and PIV, IIC showed a modestly higher discriminatory performance for predicting 360-day all-cause mortality.

These findings suggest that IIC may serve as a simple, inexpensive, and readily available biomarker for risk stratification in patients with NSTEMI. However, given its moderate predictive performance and the retrospective design of the study, IIC should be considered as an adjunctive marker rather than a replacement for established clinical risk assessment tools. Further prospective and multicenter studies are needed to validate its prognostic utility in NSTEMI populations.

## Figures and Tables

**Table 1:** Baseline clinical characteristics of the NSTEMI and Control groups.

Variable	Control Group (*n* = 1102)	NSTEMI Group (*n* = 1172)	*p*-Value
Age, years	70.30 ± 10.32	71.02 ± 13.02	0.143
Male, *n* (%)	560 (50.8)	580 (49.5)	0.527
Diabetes mellitus, *n* (%)	241 (21.9)	289 (24.7)	0.116
Hypertension, *n* (%)	610 (55.4)	687 (58.6)	0.116
Chronic kidney disease, *n* (%)	370 (33.6)	430 (36.7)	0.120

Data are presented as mean ± standard deviation or *n* (%). Continuous variables were compared using the independent-samples *t*-test, and categorical variables were compared using the Pearson chi-square test.

**Table 2:** Laboratory parameters and inflammatory indices in the control and NSTEMI groups.

Variable	Control Group (*n* = 1102)	NSTEMI Group (*n* = 1172)	*p*-Value
Leukocyte, ×10^3^/µL	7.60 ± 2.17	11.62 ± 4.35	<0.001
Neutrophil, ×10^3^/µL	4.41 ± 1.80	9.18 ± 4.27	<0.001
Lymphocyte, ×10^3^/µL	2.40 ± 0.86	1.62 ± 1.28	<0.001
Monocyte, ×10^3^/µL	0.56 ± 0.21	0.73 ± 0.41	<0.001
Hemoglobin, g/dL	14.04 ± 1.83	12.63 ± 2.26	<0.001
Platelet, ×10^3^/µL	258.12 ± 67.98	247.94 ± 83.60	0.001
RDW, %	13.20 (12.80–14.10)	14.00 (13.20–15.40)	<0.001
MCV, fL	87.09 ± 5.63	87.75 ± 6.74	0.011
AST, U/L	23 (19.00–29.00)	38.00 (21.00–124.25)	<0.001
ALT, U/L	20 (15.00–27.00)	25.00 (15.00–43.00)	<0.001
Albumin, g/dL	4.14 ± 0.37	3.44 ± 0.52	<0.001
Glucose, mg/dL	104.00 (90.00–133.00)	138.00 (110.00–199.00)	<0.001
eGFR, mL/min/1.73 m^2^	88.92 ± 22.69	71.41 ± 29.56	<0.001
Potassium, mmol/L	4.53 ± 0.51	4.28 ± 0.65	<0.001
LDH, U/L	257.00 (217.00–318.00)	293.00 (216.00–488.00)	<0.001
Sodium, mmol/L	138.08 ± 2.57	136.45 ± 3.71	<0.001
CRP, mg/dL	0.30 (0.20–0.70)	1.10 (0.40–4.00)	<0.001
NLR	1.67 (1.27–2.37)	7.23 (3.25–11.86)	<0.001
PLR	107.57 (84.81–141.96)	182.15 (110.13–286.29)	<0.001
SII	432.22 (312.87–633.77)	1701.12 (752.29–2874.68)	<0.001
PIV	234.67 (147.97–374.95)	924.02 (385.15–1930.10)	<0.001
IIC	1.90 (1.45–2.89)	9.08 (4.05–15.03)	<0.001

Data are presented as mean ± standard deviation or median (interquartile range), as appropriate. Continuous variables were compared using the independent-samples *t*-test or Mann–Whitney U test according to data distribution. RDW, red cell distribution width; MCV, mean corpuscular volume; AST, aspartate aminotransferase; ALT, alanine aminotransferase; eGFR, estimated glomerular filtration rate; LDH, lactate dehydrogenase; CRP, C-reactive protein; NLR, neutrophil-to-lymphocyte ratio; PLR, platelet-to-lymphocyte ratio; SII, systemic immune-inflammation index; PIV, pan-immune-inflammation value; IIC, Cumulative Inflammatory Index.

**Table 3:** Comparison of clinical and laboratory parameters according to mortality status in NSTEMI patients.

Variable	Survivors (*n* = 1009)	Non-Survivors (*n* = 163)	*p*-Value
Male, *n* (%)	494 (49.0)	86 (52.8)	0.275
Hypertension, *n* (%)	551 (54.6)	136 (83.4)	<0.001
Diabetes mellitus, *n* (%)	241 (23.9)	48 (29.4)	0.132
Chronic kidney disease, *n* (%)	328 (32.5)	102 (62.6)	<0.001
Age, years	70.04 ± 12.86	77.10 ± 12.38	<0.001
CK-MB mass, ng/mL	60.23 ± 89.43	49.38 ± 84.85	0.140
Troponin I, ng/mL	9.04 ± 10.52	7.49 ± 9.52	0.058
Leukocyte, ×10^3^/µL	11.45 ± 4.16	12.67 ± 5.24	0.005
Neutrophil, ×10^3^/µL	8.92 ± 4.07	10.78 ± 5.04	<0.001
Lymphocyte, ×10^3^/µL	1.71 ± 1.33	1.10 ± 0.74	<0.001
Monocyte, ×10^3^/µL	0.72 ± 0.40	0.73 ± 0.49	0.860
Hemoglobin, g/dL	12.70 ± 2.23	12.19 ± 2.42	0.007
RDW, %	13.80 (13.10–15.20)	14.60 (13.80–16.70)	<0.001
MCV, fL	87.66 ± 6.50	88.36 ± 8.09	0.292
Platelet, ×10^3^/µL	250.19 ± 79.76	234.04 ± 103.50	0.059
AST, U/L	37.00 (20.00–124.00)	45.00 (25.00–128.00)	0.069
ALT, U/L	24.00 (15.00–41.00)	29.00 (17.00–52.00)	0.003
Glucose, mg/dL	137.00 (109.00–195.00)	144.00 (113.00–211.00)	0.114
eGFR, mL/min/1.73 m^2^	73.86 ± 29.02	56.30 ± 28.49	<0.001
Potassium, mmol/L	4.25 ± 0.62	4.49 ± 0.75	<0.001
Sodium, mmol/L	136.61 ± 3.51	135.48 ± 4.65	0.004
LDH, U/L	286.00 (212.00–475.50)	329.00 (237.00–567.50)	0.001
Albumin, g/dL	3.48 ± 0.52	3.23 ± 0.48	<0.001
CRP, mg/dL	1.00 (0.40–3.40)	2.60 (0.80–7.20)	<0.001
NLR	6.73 (3.03–11.34)	10.34 (6.40–20.13)	<0.001
PLR	175.00 (107.15–278.17)	232.26 (155.00–397.53)	<0.001
SII	1600.52 (696.17–2736.02)	2300.39 (1262.71–4837.20)	<0.001
PIV	868.97 (364.42–1805.37)	1413.65 (593.83–3096.28)	<0.001
IIC	8.57 (3.73–14.06)	14.25 (8.56–26.59)	<0.001

Data are presented as mean ± standard deviation, median (interquartile range), or *n* (%), as appropriate. Continuous variables were compared using the independent-samples *t*-test or Mann–Whitney U test according to data distribution, and categorical variables were compared using the Pearson chi-square test or Fisher’s exact test, as appropriate. Abbreviations: CK-MB, creatine kinase-myocardial band; RDW, red cell distribution width; MCV, mean corpuscular volume; AST, aspartate aminotransferase; ALT, alanine aminotransferase; eGFR, estimated glomerular filtration rate; LDH, lactate dehydrogenase; CRP, C-reactive protein; NLR, neutrophil-to-lymphocyte ratio; PLR, platelet-to-lymphocyte ratio; SII, systemic immune-inflammation index; PIV, pan-immune-inflammation value; IIC, Cumulative Inflammatory Index.

**Table 4:** Univariable logistic regression analysis of predictors of 360-day all-cause mortality.

Variable	OR	95% CI for OR	*p*-Value
Age	1.045	1.030–1.060	<0.001
Male sex	1.203	0.863–1.676	0.275
Diabetes mellitus	1.372	0.952–1.976	0.089
eGFR	0.980	0.974–0.986	<0.001
Troponin I	0.990	0.974–1.006	0.234
Hemoglobin	0.912	0.847–0.981	0.013
Albumin	0.419	0.308–0.570	<0.001
CRP	1.057	1.032–1.083	<0.001
NLR	1.074	1.054–1.094	<0.001
IIC	1.058	1.043–1.073	<0.001
PIV	1.016	1.008–1.024	<0.001
SII	1.020	1.013–1.027	<0.001
PLR	1.241	1.147–1.344	<0.001

Abbreviations: OR, odds ratio; CI, confidence interval; eGFR, estimated glomerular filtration rate; CRP, C-reactive protein; NLR, neutrophil-to-lymphocyte ratio; IIC, Cumulative Inflammatory Index; PIV, pan-immune-inflammation value; SII, systemic immune-inflammation index; PLR, platelet-to-lymphocyte ratio. The odds ratios for PIV, SII, and PLR indicate the change in 360-day all-cause mortality risk per 100-unit increase in each index.

**Table 5:** Multivariable logistic regression analysis of hematological inflammatory indices for predicting 360-day all-cause mortality.

Variable	Model 1 NLR	Model 2 PIV	Model 3 SII	Model 4 IIC	Model 5 PLR
Age	1.023 (1.006–1.041); *p* = 0.007	1.028 (1.011–1.045); *p* = 0.001	1.026 (1.009–1.043); *p* = 0.002	1.022 (1.005–1.040); *p* = 0.010	1.026 (1.009–1.043); *p* = 0.003
Diabetes mellitus	1.020 (0.684–1.521); *p* = 0.922	1.148 (0.778–1.693); *p* = 0.487	1.030 (0.693–1.531); *p* = 0.882	1.033 (0.692–1.542); *p* = 0.875	1.051 (0.709–1.559); *p* = 0.803
eGFR	0.988 (0.981–0.995); *p* = 0.002	0.987 (0.980–0.994); *p* < 0.001	0.987 (0.980–0.994); *p* < 0.001	0.989 (0.981–0.996); *p* = 0.002	0.986 (0.979–0.994); *p* < 0.001
Hemoglobin	1.054 (0.966–1.150); *p* = 0.238	1.069 (0.981–1.166); *p* = 0.128	1.073 (0.985–1.170); *p* = 0.108	1.057 (0.969–1.154); *p* = 0.209	1.095 (0.987–1.194); *p* = 0.069
Albumin	0.570 (0.380–0.854); *p* = 0.007	0.578 (0.389–0.859); *p* = 0.007	0.574 (0.385–0.855); *p* = 0.006	0.591 (0.394–0.887); *p* = 0.011	0.588 (0.396–0.874); *p* = 0.009
CRP	0.994 (0.962–1.027); *p* = 0.715	1.011 (0.981–1.042); *p* = 0.481	1.006 (0.975–1.037); *p* = 0.727	0.994 (0.962–1.028); *p* = 0.741	1.011 (0.981–1.043); *p* = 0.466
NLR	1.058 (1.037–1.080); *p* < 0.001	-	-	-	-
PIV	-	1.010 (1.002–1.018); *p* = 0.017	-	-	-
SII	-	-	1.015 (1.008–1.022); *p* < 0.001	-	-
IIC	-	-	-	1.045 (1.029–1.060); *p* < 0.001	-
PLR	-	-	-	-	1.163 (1.066–1.269); *p* = 0.001

Values are presented as adjusted odds ratios (95% confidence interval). Separate multivariable logistic regression models were constructed for each hematological inflammatory index to avoid multicollinearity among mathematically related indices. Each model was adjusted for age, diabetes mellitus, eGFR, hemoglobin, albumin, and CRP. NLR and IIC were analyzed per 1-unit increase, whereas PIV, SII, and PLR were analyzed per 100-unit increase.

**Table 6:** ROC analysis and optimal cut-off values of hematological inflammatory indices for predicting 360-day all-cause mortality.

Variable	AUC	95% CI	*p*-Value	Optimal Cut-Off	Sensitivity	Specificity	Youden Index
IIC	0.704	0.661–0.746	<0.001	≥8.20	81.5%	67.9%	0.494
NLR	0.676	0.632–0.720	<0.001	≥6.93	72.2%	51.2%	0.234
SII	0.636	0.589–0.682	<0.001	≥2203	52.5%	65.0%	0.175
PIV	0.621	0.574–0.668	<0.001	≥1304	54.3%	65.4%	0.197
PLR	0.619	0.572–0.667	<0.001	≥170	71.0%	48.9%	0.199

Abbreviations: ROC, receiver operating characteristic; AUC, area under the curve; CI, confidence interval; IIC, Cumulative Inflammatory Index; NLR, neutrophil-to-lymphocyte ratio; SII, systemic immune-inflammation index; PIV, pan-immune-inflammation value; PLR, platelet-to-lymphocyte ratio. Optimal cut-off values were determined using the Youden index.

**Table 7:** Pairwise DeLong comparisons of AUC values for predicting 360-day all-cause mortality.

Comparison	AUC Difference	z Value	*p*-Value
IIC vs. NLR	0.028	3.691	<0.001
IIC vs. SII	0.067	5.169	<0.001
IIC vs. PIV	0.083	3.671	<0.001
IIC vs. PLR	0.084	4.750	<0.001

AUC, area under the curve; IIC, Cumulative Inflammatory Index; NLR, neutrophil-to-lymphocyte ratio; SII, systemic immune-inflammation index; PIV, pan-immune-inflammation value; PLR, platelet-to-lymphocyte ratio.

## Data Availability

The data supporting the findings of this study are available from the corresponding author upon reasonable request, subject to institutional and ethical restrictions.

## Supplementary Materials

The following supporting information can be downloaded at https://www.mdpi.com/article/10.3390/biomedicines14071415/s1, Table S1: Goodness-of-fit statistics for multivariable logistic regression models.

## Abbreviations

The following abbreviations are used in this manuscript:

ACS	acute coronary syndrome
ALT	alanine aminotransferase
AST	aspartate aminotransferase
AUC	area under the curve
CAD	coronary artery disease
CI	confidence interval
CK-MB	creatine kinase-myocardial band
CRP	C-reactive protein
eGFR	estimated glomerular filtration rate
IIC	Cumulative Inflammatory Index
LDH	lactate dehydrogenase
MCV	mean corpuscular volume
NLR	neutrophil-to-lymphocyte ratio
NSTEMI	non-ST-segment elevation myocardial infarction
OR	odds ratio
PIV	pan-immune-inflammation value
PLR	platelet-to-lymphocyte ratio
RDW	red cell distribution width
ROC	receiver operating characteristic
SII	systemic immune-inflammation index
