# Supplementary material for: Prognostic Value of the Cumulative Inflammatory Index (IIC) in Patients with Non-ST-Segment Elevation Myocardial Infarction
Source: Biomedicines. 2026 Jun 23;14(7):1415. doi: 10.3390/biomedicines14071415 (PMC13405717; doi:10.3390/biomedicines14071415)
Supplement: Supplementary file 1 [file biomedicines-14-01415-s001.zip › biomedicines-4357458-supplementary.pdf]

Table S1. Goodness-of-fit statistics for multivariable logistic regression models.

**Model**	**Index**	**-2 Log Likelihood**	**Nagelkerke R^2**	**Hosmer–Lemeshow χ^2**	***p* Value**
Model 1	NLR	810.538	0.193	6.307	0.613
Model 2	PIV	846.754	0.142	7.705	0.463
Model 3	SII	823.720	0.174	2.095	0.978
Model 4	IIC	790.148	0.221	8.115	0.422
Model 5	PLR	835.350	0.158	4.390	0.820
